# The occurrence of acute primary angle closure triggered, aggravated, and accelerated by COVID-19 infection: retrospective observational study

**DOI:** 10.3389/fpubh.2023.1196202

**Published:** 2023-08-14

**Authors:** Yue Ying, Ruyi Zhai, Yanan Sun, Qilian Sheng, Xintong Fan, Xiangmei Kong

**Affiliations:** ^1^Eye Institute and Department of Ophthalmology, Eye & ENT Hospital, Fudan University, Shanghai, China; ^2^NHC Key Laboratory of Myopia, Key Laboratory of Myopia, Chinese Academy of Medical Sciences, Fudan University, Shanghai, China; ^3^Shanghai Key Laboratory of Visual Impairment and Restoration, Shanghai, China

**Keywords:** acute primary angle closure, COVID-19, ultrasound biomicroscope, SARS-COV-2, pathogenesis, elevated intraocular pressure

## Abstract

**Introduction:**

The aim of this study is to demonstrate the relevance of primary acute angle closure (APAC) and COVID-19 infection, compare the demographic features and manifestations between COVID-19 positive and negative patients with APAC, and infer the underlying mechanism.

**Methods:**

This study is based on all patients diagnosed with APAC at the glaucoma center of Eye, Ear, Nose and Throat Hospital of Fudan University (Fenyang road center) from 15th December 2022 to 11th January 2023. Totally 171 APAC cases were categorized into COVID-19 positive and negative group. Demographic features and final treatment level of the patients were compared between the two groups. Clinical manifestations, intraocular pressure, and anterior chamber configuration were also compared between the two groups.

**Results:**

In the COVID-19 positive group, the number of cases with APAC onset spiked in 22nd December 2022, which coincided with the spike of COVID-19 antigen positive people. Compared to the COVID-19 negative group, COVID-19 positive APAC patients were younger with a lower percentage of APAC history. Additionally, more eyes of COVID-19 positive APAC patients showed keratic precipitates. COVID-19 positive eyes had significantly larger anterior chamber depth with a more dilated pupil. Therefore, COVID-19 infection could probably act as a triggering factor of APAC

**Discussion:**

The onset of APAC might be accelerated by COVID-19 infection for patients with younger age and milder anatomical configuration. Additionally, COVID-19 related APAC cases might have a more abrupt and fierce onset. Ophthalmic emergent services should not be neglected during the epidemic period.

## 1. Introduction

Acute primary angle-closure (APAC) is a pathologic status characterized by sudden elevation of intraocular pressure (IOP) due to abrupt closure of the anterior chamber angle. Patients may experience headache, eye pain, nausea, vomiting, and loss of vision. If not controlled timeously, the markedly elevated IOP can cause irreversible optic nerve damage and visual field loss, as observed in primary angle-closure glaucoma (PACG). Therefore, APAC is an emergent ophthalmic condition causing necessary concerns.

Anatomic configuration such as small cornea, shallow anterior chamber depth, short axial length, narrow anterior chamber angle, and exaggerated lens vault are the anatomical risk factors associated with APAC. Previous studies had classified such causative factors of APAC into four anatomic levels, namely the iris level (pupillary block), the ciliary level (plateau iris), the lens level (exaggerated lens vault), and the retro-lenticular level (1). However, a combined anatomic mechanism involving cross-level factors was found in some cases of APAC (2). Apart from the pre-existing anatomic factors, sudden presence of contributing factors such as stress, strong emotion, prone position, and staying in dark room may trigger the outburst of an APAC crisis (3, 4). The underlying molecular biological mechanisms of such triggering factors might involve the neuroendocrine system (5), psychological factors (6), among others. In the Asian population, a characteristically high incidence rate of plateau iris was reported in PACG cases (7), indicating mechanism differences in different populations. The disparity in disease spectrum also lies in the prevalence, as the incidence rate of APAC in Asian people was reportedly higher when compared to the Caucasian population (8–10).

Previously, several case reports described APAC cases in patients with Coronavirus Disease 2019 (COVID-19), and the correlation between COVID-19 infection and APAC onset was strongly suggested (11–13). However, there has been a lack of studies including a large-sample size of COVID-19 in patients with APAC. In December 2022, after implementing “dynamic zero COVID” policy for 3 years, China’s lifting of all such restrictions led to a soaring number of COVID-19 cases countrywide. Therefore, a large majority of the country’s medical resources was fed into the treatment of COVID-19 patients. Our center, as a tertiary hospital specialized in ophthalmology, undertook most of the emergent ophthalmic cases in Shanghai. During the outbreak, a lot more patients in our center were diagnosed of APAC than the former years, yet their demographic features, manifestations, and correlation with COVID-19 was not known.

In this retrospective study, medical record of all APAC cases diagnosed within 1 month after the termination of “dynamic zero COVID” policy in our center was studied. This study aimed to demonstrate the characteristics of APAC cases in Shanghai from 15th December 2022 to 11th January 2023, compare the clinical features between SARS-COV-2 positive and negative cases, and probe into the role of COVID-19 infection in APAC pathogenesis.

## 2. Materials and methods

### 2.1. Study design and participants

This study is a retrospective observational study based on outpatient medical records of all patients with APAC at the glaucoma center of Eye, Ear, Nose, and Throat (EENT) Hospital of Fudan University (Fenyang road center), Shanghai, China. The study period was within 1 month after China’s relaxation from “dynamic zero COVID” policy, from 15th December 2022 to 11th January 2023. The study followed the ethical standards of the Declaration of Helsinki and was approved by the ethics committee of Eye & ENT Hospital, Fudan University.

The diagnosis of APAC was clarified if the patients met the following criteria: (I) at least two of the following symptoms: pain in ocular area, nausea or vomiting, and history of visual blurring or loss, (II) elevated IOP, (III) at least three of the following signs: conjunctival congestion, corneal edema, dilated or irregular-shaped unreactive pupil, and shallow anterior chamber depth, and (IV) presence of angle-closure configuration. The exclusion criteria included: (I) lack of ultrasound biomicroscope (UBM) result in our center, (II) angle-closure secondary to other ophthalmic conditions, and (III) lack of information on whether the patient had COVID-19 infection history before or after the APAC onset from medical record or telephonic follow-up.

**Figure 1:**
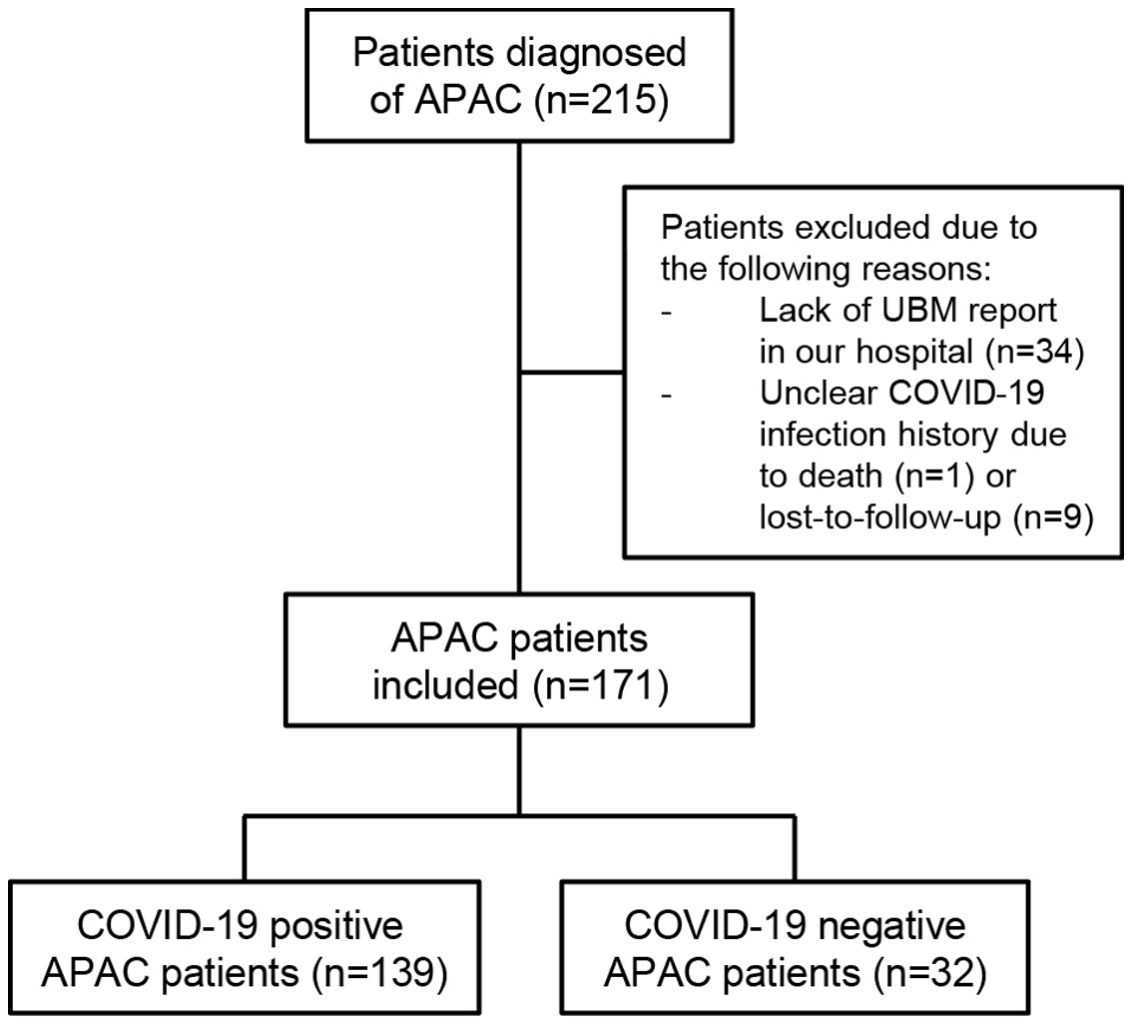
The flow diagram of patient inclusion.

Totally 171 patients met the up-mentioned criteria and were categorized into COVID-19 positive APAC group and COVID-19 negative APAC group. The patient flow diagram is shown in Figure 1. The COVID-19 positive patients were defined as patients whose APAC symptoms started 2 days before the onset of COVID-19 symptoms or within 5 days after COVID-19 onset. The onset of COVID-19 was defined as the date when typical symptoms showed up for those with contact history with diagnosed patients, or the date when they had positive COVID-19 antigen test for those who were asymptomatic. Patients without COVID-19 infection or with COVID-19 infection out of the above-mentioned time range were categorized into the COVID-19 negative group. Among the included participants, 21 patients had bilateral APAC attack. Because 7 unilaterally affected patients only did UBM examination for the affected eyes, 192 affected eyes and 143 fellow eyes were included.

### 2.2. Data collection

Demographic features were collected from the medical records of the patients, including age, gender, APAC eye, past history of APAC attack, family history of glaucoma, date of APAC attack, and chief complaint. Maximal IOP was defined as the highest IOP during the attack phase. If the patient had been treated at another hospital, their maximal IOP was acquired from telephonic follow-up. For those patients whose IOP once exceeded the measuring range, the maximal IOP was recorded as 60 mmHg. Clinical signs including conjunctiva/ciliary congestion, cornea edema, keratic precipitates (KP), anterior chamber depth and inflammation, and pupil configuration were obtained from slit-lamp examination record. UBM results during the attack were also documented. The patients’ highest required treatment was categorized into medication, laser, or surgery. COVID-19 infection history including date of onset and their use of medicine before APAC onset was recorded from telephonic follow-up. The follow-up was conducted from 31st January 2023 to 19th February 2023.

### 2.3. Statistical and data analysis

Statistics were analyzed using STATA 16.0 (College Station, TX, United States). Normally and non-normally distributed data were expressed using mean ± standard deviation and median (P25, P75), respectively. Student’s *t* test was used to compare the differences in normally distributed quantitative data. Wilcoxon signed rank sum test was used to compare the differences in non-normally distributed quantitative data and qualitative data. Pearson chi-square test was used in comparing categorical data. *p* value under 0.05 was considered statistically significant.

## 3. Results

### 3.1. Number of patients and correlation with COVID-19 outbreak

**Figure 2:**
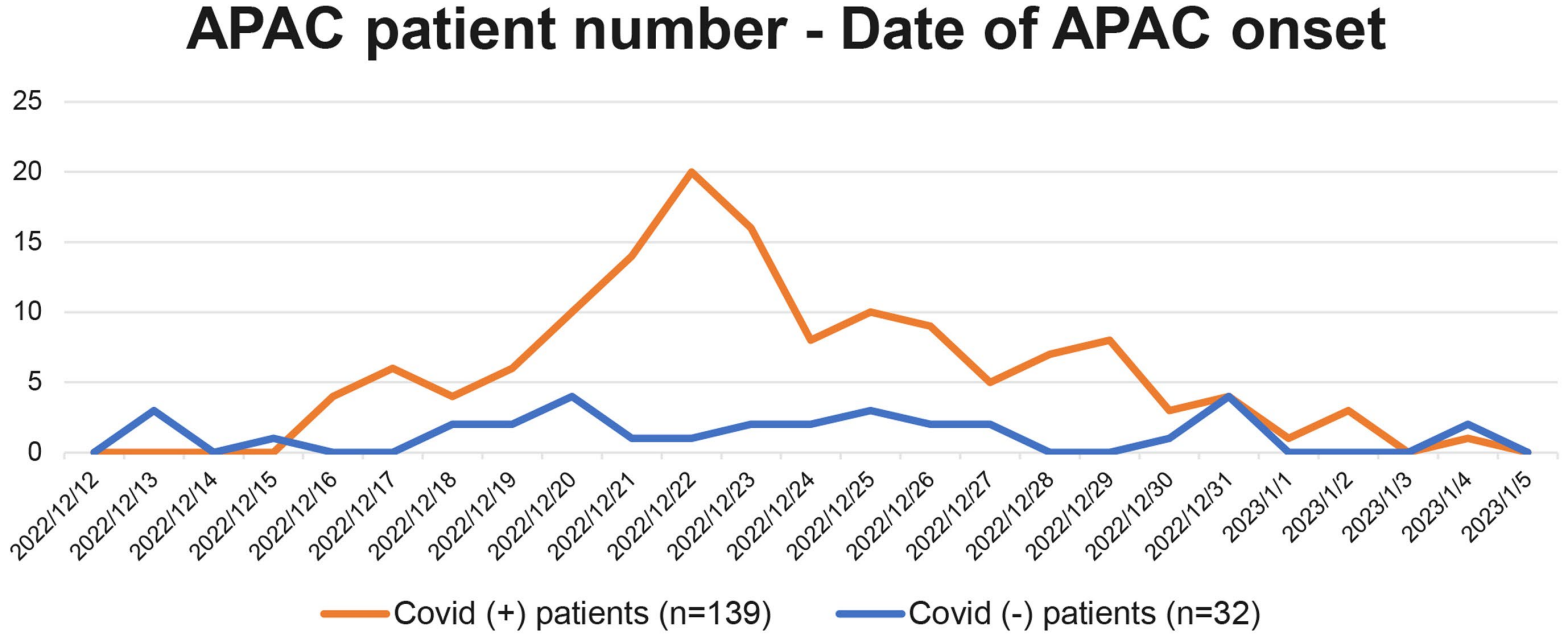
Correlation between the number of patients with APAC and their date of APAC onset. The orange line represents all 139 APAC in COVID-19 positive group from 2022/12/15 to 2023/1/11. The blue line represents the 32 patients in COVID-19 negative group.

**Figure 3:**
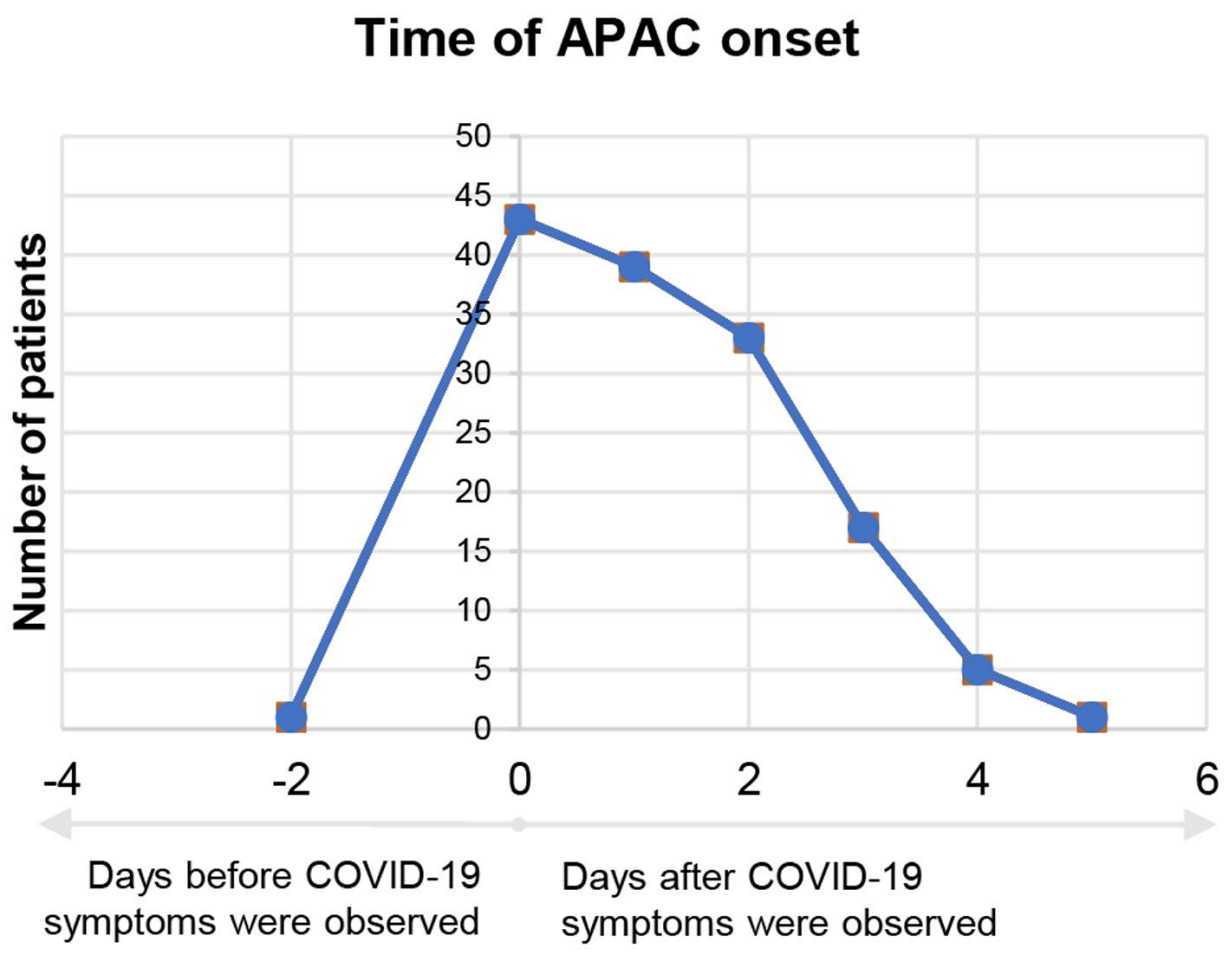
Time interval between APAC onset and start of COVID-19 symptoms of COVID-19 positive group. Negative number in the horizontal axis means APAC onset before COVID-19 symptoms appeared. Positive number in the horizontal axis means APAC onset after COVID-19 symptoms appeared.

Correlation between the number of APAC patients and date of their APAC onset is demonstrated in Figure 2. The number of COVID-19 negative APAC patients remained relatively stable over the time (blue line, Figure 2). However, the number of COVID-19 positive APAC patients dramatically increased after 15th December 2022, and spiked on 22nd December 2022 (21 patients). Then, the number of APAC patients gradually declined with fluctuation. Two minor spikes appeared on 25th December 2022 and 29th December 2022. The number of patients with APAC returned to baseline after 3rd January 2023. This trend is similar to the data of COVID-19 antigen from the Chinese Center for Disease Control and Prevention (CCDC) COVID-19 clinical and surveillance Data, as it also spiked on 22nd December 2022, fluctuated and declined with minor spikes on 26th December 2022 and 5th January 2023 (14). Figure 3 displays the time interval between APAC onset and COVID-19 onset. 83.45% (116/139) of the patients felt the symptoms of APAC on the same day or within 2 days after COVID-19 onset.

### 3.2. Demographic features of study participants

Among the 171 APAC patients, 139 patients reported COVID-19 infection history. Patients in COVID-19 positive group were significantly younger than the COVID-19 negative group (63.50 ± 8.28 vs. 66.31 ± 7.64, *p* = 0.040). There were more females in both COVID-19 positive group (66.19%) and COVID-19 negative group (68.75%) compared to males. Significantly less patients from the COVID-19 positive group had a past APAC attack (15.83% vs. 31.25%, *p* = 0.044). The percentage of patients who had bilateral attack and patients with family history of glaucoma were comparable between the two groups. Additionally, 43.17% (60/139) of the patients used NSAIDs orally before APAC onset due to COVID-19 symptoms. Another 7 patients (5.04%) used antiviral medicine and 4 patients (2.88%) used antibiotics. Highest level of required treatment was comparable between groups. Table 1 showed the demographic features and required treatment for APAC patients.

**Table 1:** Features of COVID-19 positive and COVID-19 negative APAC patients.

	COVID-19 (+) patients (*n* = 139)	COVID-19 (−) patients (*n* = 32)	*p* value
Demographic Characteristics			
Age (years)	63.50 ± 8.28	66.31 ± 7.64	0.040*
Sex (Male/Female)	47/92	10/22	0.782
Affected eye			0.749
OD	60	16	
OS	62	12	
OU	17	4	
Past history of APAC (+/−)	22/117	10/22	0.044*
Family history of glaucoma (+/−)	27/112	7/25	0.754
Systematic use of medication before APAC onset			
NSAIDs	60/139	/	
Antiviral treatment	7/139	/	
Antibiotics	4/139	/	
Final required treatment solution			0.692
Antiglaucoma eyedrops	5	0	
Laser	28	9	
Surgery	106	23	

Datas are expressed as mean ± SD or number of patients. APAC, acute primary angle closure; OD, oculus dexter; OS, oculus seminar; OU, oculus uterque; NSAIDs, non-steroidal anti-inflammatory drugs. *p < 0.05.The bold values were emphasized because these were statistically significant (*p* value < 0.05).

### 3.3. Clinical manifestations and anterior chamber configuration

The clinical manifestations and anatomical configuration of APAC affected eyes are demonstrated in Table 2. More APAC eyes from COVID-19 positive group showed KP than COVID-19 negative eyes (30.13% vs. 13.89%, *p* = 0.048). The number of patients with conjunctival/ciliary congestion, cornea edema, dilated/unreactive pupil, anterior chamber inflammation (cell and Tyndall effect) was comparable between the COVID-19 positive and negative APAC eyes. Additionally, the maximal IOP during this APAC attack was comparable between groups.

**Table 2:** Manifestations and anatomical configuration of eyes of COVID-19 positive and negative APAC patients.

	APAC affected eyes (*n* = 192)		
	COVID-19 (+) eyes (*n* = 156)	COVID-19 (−) eyes (*n* = 36)	*p* value
Slit lamp examinations			
Conjunctival/ciliary congestion (+/−)	122/34	28/8	0.955
Cornea edema (+/−)	128/28	29/7	0.834
Dilated/unreactive pupil	143/13	30/6	0.131
KP (+/−)	47/109	5/31	0.048*
Anterior chamber cell (+/−)	150/6	35/1	0.758
Anterior chamber Tyndall effect (+/−)	119/37	32/4	0.096
IOP maximum (mmHg)	47.24 ± 9.16	47.69 ± 8.77	0.405
UBM of affected eyes			
Pupil diameter (mm)	4.75 ± 1.17	4.24 ± 1.03	0.009*
Anterior chamber depth (mm)	1.66 ± 0.25	1.57 ± 0.25	0.028*
Plateau iris			0.626
None	55	12	
Mild	10	6	
severe	91	18	
Forward iris attachment location	153/3	34/2	0.217
Ciliary process pronation			
none	2	2	0.687
mild	51	11	
severe	103	23	
Exaggerated lens vault (+/−)	29/127	6/30	0.788
Loose ciliary zonule (+/−)	49/107	9/27	0.450

Datas are expressed as mean ± SD or number of patients. APAC, acute primary angle closure; KP, keratic precipitates; IOP, intraocular pressure; UBM, ultrasound biomicroscope. *p < 0.05.The bold values were emphasized because these were statistically significant (*p* value < 0.05).

The anterior chamber configuration in UBM examinations showed significantly larger pupil diameter in COVID-19 positive eyes (4.75 ± 1.17 vs. 4.24 ± 1.03, *p* = 0.009) as well as a significantly deeper anterior chamber depth (1.66 ± 0.25 vs. 1.57 ± 0.25, *p* = 0.028), although APAC eyes in both groups demonstrated dilated pupil and shallow anterior chamber depth. Other anterior chamber anatomical structures, namely plateau iris, location of iris root attachment, ciliary process configuration, lens vault, and zonule configuration, were all comparable between COVID-19 positive and negative APAC eyes. No significant difference in anatomical structures was found in the contralateral eyes between COVID-19 positive and negative group (Table 3).

**Table 3:** Anterior chamber configuration of COVID-19 positive and negative contralateral eyes.

	Contralateral eyes (*n* = 143)		
	COVID-19 (+) eyes (*n* = 115)	COVID-19 (−) eyes (*n* = 28)	*p* value
UBM of affected eyes			
Pupil diameter (mm)	2.67 ± 0.96	2.86 ± 0.76	0.325
Anterior chamber depth (mm)	1.78 (1.64, 1.95)	1.65 (1.53, 1.88)	0.062
Plateau iris			0.828
none	17	3	
mild	9	3	
severe	89	22	
Forward iris attachment point	112/3	26/2	0.243
Ciliary process pronation			
none	1	0	0.682
mild	39	11	
severe	75	17	
Exaggerated lens vault (+/−)	15/100	5/23	0.510
Loose ciliary zonule (+/−)	28/87	5/23	0.465

Datas are expressed as mean ± SD, median (P25, P75) or number of patients. UBM, ultrasound biomicroscope.

## 4. Discussion

Our study demonstrated the number of patients who had an onset of APAC symptoms within approximately 1 month after China’s relaxation from “zero-COVID” policy. Based on the same diagnosis criteria (symptoms, signs, IOP and UBM results at our center), only 12 patients were diagnosed of APAC during the same period in the previous year (15th December 2021 to 11th January 2022) at our center. Such distinction can partly be attributable to the availability for APAC patients to visit other comprehensive hospitals during COVID-19 sporadic season. Since the curve of COVID-19 positive group was quite similar to the curve of COVID-19 antigen positive people released by CCDC, COVID-19 infection was possibly a triggering factor in the pathogenesis of APAC. Notably, the number of COVID-19 negative APAC patients remained stable during our chosen time range. Most of the participants had APAC onset within 2 days after the onset of COVID-19 symptoms. In fact, 31.65% (44/139) patients described having ocular symptoms prior to or simultaneously with fever, which was also persuasive evidence of COVID-19 infection being related to APAC.

Moreover, significant differences were found between the demographic characteristics of COVID-19 positive and COVID-19 negative APAC patients. Fewer patients from COVID-19 positive group had a history of APAC attack and were younger compared to the COVID-19 negative group. Such findings indicated that COVID-19 infection might act as a strong stressor and accelerated the onset of APAC. This could be corroboratively proved by the fact that the eyes of COVID-19 positive patients showed a significantly larger anterior chamber depth. Without the COVID-19 infection, some patients might still be able to live in symptom-free state for a while despite their predisposition due to risky anatomical configuration, and such preclinical phases could be potential therapeutic windows for preventive treatment such as laser peripheral iridotomy.

All patients whose maximal IOP was once too high to be reported with an exact number was documented as 60 mmHg. Additionally, since some patients were already treated with intravenous mannitol before their first IOP test at another hospital, the maximal IOP recorded was lower than the actual value. COVID-19 positive patients with APAC had eyes with a larger pupil diameter under UBM examinations. More eyes in COVID-19 positive group showed pigmentary KP, a sign for iris atrophy. Both larger pupil diameter and more cases with punctate KP could indicate that APAC eyes from COVID-19 positive group experienced a more aggressive attack phase. Therefore, the iris tissue of COVID-19 positive patients might suffer from severer ischemia and thereafter present atrophy that is more obvious.

Previous study discovered ACE2 receptor in conjunctiva (15), which is the entry receptor of SARS-COV-2 (16). Additionally, various studies in COVID-19 infected patients worldwide showed the existence of ocular manifestations such as conjunctival hyperemia, and conjunctival discharge, etc. (17). Other studies suggested ocular symptoms could present even earlier than respiratory symptoms (18–20). Positive conjunctival swab PCR test was found in 3.9% of patients with COVID-19 (17). All such evidence proved that eyes can be affected by COVID-19 infection. Since no significant difference was found in the anatomical mechanism, the correlation between COVID-19 infection and early-onset aggressive APAC attack might be due to both systematic and local triggers.

Early studies proposed the possibility of APAC being caused by surgery and general anesthesia (21, 22). Although muscle relaxants may play a part, such reports still suggested the possible role of emotional upset and stress as triggering factors. In COVID-19 cases, infection and quarantine could also cause stress condition and negative emotions. Secondly, systematic medication was also reported as suspected APAC trigger (23), while in our study, 43.17% patients recalled using cold medication (containing NSAIDs, antihistamines) to relieve COVID-19 symptoms. Thirdly, a previous study reported a higher ocular surface temperature in glaucomatous eyes (24). In our study, 82 patients (58.99%) started to feel eye pain or vision loss concurrently with the onset of hyperthermia, or on the second day of fever. Such time consistency suggested that hyperthermia may cause potential congestion and edema in anterior chamber structures, making the narrow anterior chamber even worse. Lastly, patients with COVID-19 may spend long time relaxing in dark room, which can cause dilated pupils, thus pose a threat to the already-jammed anterior chamber angle.

Therefore, stress, depression, oral medication, fever, body position, and dark environment might contribute to APAC pathogenesis as systemic triggers.

Local ocular triggers caused by COVID-19 infection may also contribute to APAC onset. Previously, various case reports showed the possibility of uveitis and optic neuritis being related to COVID-19 infection (19, 25–27). Moreover, SARS-COV-2 was detected in the aqueous humour of asymptomatic COVID-19 patients (28). Despite the low detection rate, these studies suggested that apart from the ocular surface, intraocular involvement could also be possible during COVID-19 infection. However, low positive rate also suggested that in most cases, SARS-COV-2 did not interfere with the intraocular environment by directly entering the eyes. Changes in ocular microenvironment might contribute to the pathogenesis and prognosis of APAC. Various early studies in PACG showed changes in aqueous humour immune microenvironment (29, 30) and iris immune status (31), which may lead to a local immune disorder. Additionally, intraocular microenvironment may also be interfered by the blood supply as increased retinal vessel diameter during COVID-19 infection and decreased vessel diameter after remission were reported (32), as well as some cases of retinal vein occlusion (33–35). Reduced vessel density and enlarged fovea avascular zone were found in COVID-19 infected patients (36), and were explained as the ocular consequence of systematic thrombotic microangiopathy and hypercoagulation (37). Therefore, changes in immune and vascular microenvironment may be local ocular factors and trigger an all-or-none change in anterior chamber angle. However, the explicit relationship between viral infection and APAC onset still requires further studies.

This study had several limitations. Firstly, although this study included a large number of patients with APAC, patients from the other branches of EENT Hospital, Fudan University as well as patients who did not visit the glaucoma center were not included. Additionally, 10 patients’ COVID-19 infection history was unclear because of death or lost-to-follow-up. Second, for patients who reported their COVID-19 infection history and maximal IOP, recalling bias was unavoidable because the follow-up was conducted approximately 1 month after their first visit. Thirdly, as reported by CCDC, the main virus subvariant affecting Shanghai during the outbreak was BA5.2 (14). With a high vaccination coverage, this mild strain of SARS-COV-2 may bring about a high percentage of asymptomatically infected patients. Some patients were categorized into COVID-19 negative group simply because they had no symptom and they did not test for COVID-19. Therefore, the difference between groups might have be underestimated. Lastly, longer follow-up time is needed to understand differences in long-term prognosis of COVID-19 positive and negative APAC patients.

In conclusion, this is the first large-sample study of COVID-19 and APAC. Relevance between APAC attack and COVID-19 infection was highly suspected. COVID-19 infection accelerated the occurrence of APAC since infected patients were younger, had milder anatomical malformation and less possibility of past APAC attacks. COVID-19 positive relevant APAC patients might have experienced a more abrupt attack phase. This study highlights the importance of medical resource allocation to emergent ophthalmic cases, even during epidemic period of infectious diseases in the future.

## Data availability statement

The raw data supporting the conclusions of this article will be made available by the authors, without undue reservation.

## Ethics statement

The studies involving human participants were reviewed and approved by the ethics committee of Eye & ENT Hospital, Fudan University. Written informed consent for participation was not required for this study in accordance with the national legislation and the institutional requirements.

## Author contributions

YY contributed to manuscript writing, data analysis, and conceptualization. RZ contributed to conceptualization and supervision. YS, QS, and XF participated in data collection and input. XK designed and guided the whole study. All authors contributed to the article and approved the submitted version.

## Funding

This study was supported by the Western Medicine Guidance Project of Shanghai Committee of Science and Technology (19411961600), the Experimental Animal Research Project of Shanghai Science and Technology (201409006600), and the Double Excellent Project of EENT Hospital (SYB202003). The authors were funded by the Surface Project of National Natural Science Foundation of China (81770922 and 82070957). The funders had no role in study design, data collection, analysis, and interpretation, decision to publish, or preparation of the manuscript.

## Conflict of interest

The authors declare that the research was conducted in the absence of any commercial or financial relationships that could be construed as a potential conflict of interest.

## Publisher’s note

All claims expressed in this article are solely those of the authors and do not necessarily represent those of their affiliated organizations, or those of the publisher, the editors and the reviewers. Any product that may be evaluated in this article, or claim that may be made by its manufacturer, is not guaranteed or endorsed by the publisher.
